# The Impact of Curviness on Four Different Image Sensor Forms and Structures

**DOI:** 10.3390/s18020429

**Published:** 2018-02-01

**Authors:** Wei Wen, Siamak Khatibi

**Affiliations:** Department of Technology and Aesthetics, Blekinge Tekniska Högskola, 37141 Karlskrona, Sweden; siamak.khatibi@bth.se

**Keywords:** software-based, virtual, hexagonal image, grid structure, pixel form, fill factor, curviness quantification, Hessian matrix, critical points

## Abstract

The arrangement and form of the image sensor have a fundamental effect on any further image processing operation and image visualization. In this paper, we present a software-based method to change the arrangement and form of pixel sensors that generate hexagonal pixel forms on a hexagonal grid. We evaluate four different image sensor forms and structures, including the proposed method. A set of 23 pairs of images; randomly chosen, from a database of 280 pairs of images are used in the evaluation. Each pair of images have the same semantic meaning and general appearance, the major difference between them being the sharp transitions in their contours. The curviness variation is estimated by effect of the first and second order gradient operations, Hessian matrix and critical points detection on the generated images; having different grid structures, different pixel forms and virtual increased of fill factor as three major properties of sensor characteristics. The results show that the grid structure and pixel form are the first and second most important properties. Several dissimilarity parameters are presented for curviness quantification in which using extremum point showed to achieve distinctive results. The results also show that the hexagonal image is the best image type for distinguishing the contours in the images.

## 1. Introduction

The arrangement and form of photoreceptors vary from the fovea to the periphery of the retina. This is a consequence of evolution which argues that the arrangement and form of camera pixel sensors should be variable as well. However practical issues and history of camera development have made us to use fixed arrangements and forms of pixel sensors. Our previous works [1,2] showed that despite the limitations of hardware, it is possible to implement a software method to change the size of pixel sensors. In this paper, we present a software-based method to change the arrangement and form of pixel sensors.

The pixel sensor arrangement is often referred to as the grid structure. Most available cameras have rectangular grid structures. Previous works [3,4] have shown the feasibility of converting the rectangular to hexagonal grid structure by a half pixel shifting method (i.e., a software-based approach). Generation of the hexagonal pixel form is generally achieved by interpolation of intensity values of the rectangular pixel form. In this paper, we present a method, based on our previous works, for maximizing the size of rectangular pixel forms, that generates a hexagonal pixel form on a hexagonal grid. Each original rectangular pixel form is deformed to a hexagonal one using modelling of the incident photons onto the senor surface. To the best of our knowledge, there is no previous method which can offer hexagonal deformation of pixel form on a hexagonal grid.

The comparison of different grid structures or different pixel forms is a challenging task and should be directed to a more specific task. Inasmuch as human vision is highly evolved to detect objects in a dynamic natural scene, the gradient computation as an elementary operation in object detection becomes interesting and appropriate candidate for this specific task. We have focused our investigation on the effect of the sharp transitions in the contour of objects which is estimated by first and second order of gradient computation on the images generated by different grid structures and different pixel forms. Two categories of images having curved versus linear edges of the same object in a pair of images, are used to estimate the detectability of each of the four considered sensor structures for curviness.

This paper is organized as follows: in Section 2, related research on hexagonal grid resampling and form is explained. Then the four types of image generations are explained in Section 3. Section 4 and Section 5 present the methodology of curviness quantification and the experiment setup, respectively, then the results are shown and discussed in Section 6. Finally, we summarize the work described in this paper in Section 7.

## 2. Background

Due to a higher sampling efficiency, consistent connectivity and higher angular resolution of hexagonal grids [3] in comparison to square grids, in the last four decades hexagonal grids have been investigated in numerous methods and applications [3,5,6,7], which include image reconstruction [5], edge detection [7,8], image segmentation [9], and motion estimation [10]. Different algorithms and mathematical models have emerged in recent years to acquire hexagonal grids. For example, the rectangular grid can be suppressed in rows and columns alternatively and be sub-sampled; i.e., by a half-pixel shifting method [11]. In this way, a bigger hexagonal pixel is generated at the cost of obtaining lower resolution in comparison to the original rectangular grid. In the method, the distance between rows is changed by $\sqrt{3}/2$ and the pixel shifting can be achieved e.g., by implementing normalized convolution [12]. The significances of such a structure are the equidistant and 60 degrees intersection of the sampling points. In Yabushita et al. [5], the pseudohexagonal elements are composed of small square pixels with an aspect ratio of 12:14, which was later implemented by Jeevan et al. with a different ratio of 9:8 [13]. In the spiral architecture of He et al. [14] four square pixels are averaged and generate a hexagonal pixel. Based on the spiral architecture, a design procedure for the development of hexagonal tri-directional derivative operators is present in [15], that can be applied directly to hexagonal images and can be used to improve both the efficiency and accuracy with respect to feature extraction on conventional intensity images. Although the architecture preserves the main properties of object, it loses some degree of resolution, which has an impact, especially on the result of edge detection applications [8]. Later this architecture was improved by Wu et al. [16], by mapping the rectangular grids to hexagonal ones, processing images on hexagonal grids, and remapping the results to the square grids. By processing images on a hexagonal grid less distortion was observed [3]. All above software-based methods have one major common property: they convert the rectangular grid to the hexagonal one using a linear combination of rectangular pixels. The technique of resampling digital images on this pseudohexagonal grid by using three interpolation kernels is proposed in [17] and one blurring kernel have been demonstrated. Then a new spline based on a least-squares approach is presented in [18], used for converting to a hexagonal lattice and has been demonstrated to achieve better quality than traditional interpolative resampling. In the most recent research, Ref [19] introduced a method to convert images from square to hexagonal lattices in the frequency domain using the Fourier transform. However, in our approach, the rectangular pixels initiate a non-linear learning model based on the photons incident onto the senor surface. The approach is based on our earlier works [20,21] where the fill factor of an arbitrary image was estimated and used to obtain an enhanced image, as captured by a 100% fill factor sensor.

Recently as image quality and preserving of the quality during operations such as translation, rotation, and super resolution have become important issues to the research community, implementing a hexagonal grid has been shown to be one of the alternative solutions [12,22]. The hexagonal grid is our visual system solution for observing our complex environmental scenes. Believing that such natural scenes have had a great impact on the evolution of our visual system; i.e., in the creation of hexagonal grid in the fovea of the retina, then the justified question is which features in a natural scene and in its dynamical alteration cause such an impact. The pioneering work was done by Gestalt psychologists and, more in detail, by Rubin [23], who first demonstrated that contours contain most of the information related to object perception, like the shape, the color and the depth. In fact, by investigating simple conditions like those used by Gestalt psychologists, mostly consisting of contours only, Pinna et al. [24] demonstrated that the phenomenal complexity of the material attributes emerges through appropriate manipulation of the contours. Bar et al. [16] showed in their psychological study that our visual system prefers curved visual objects; i.e., the physical property of objects in a scene, which is manifested in sharp transitions in the contour of objects, has a critical influence on our perception of that object. Other studies such as [25] show the capacity of the hexagonal grid on detection of the sharp transitions in contour of objects. In this paper, we implement the curviness; i.e., the sharp transitions in contour of objects, as a comparison feature to evaluate four different grid structures.

## 3. Image Generation

In this section, we explain the generation of hexagonal enriched, square enriched, haft pixel shift enriched, and half pixel shift images from an original image which has a square pixel form on a square grid.

### 3.1. Generation of the Hexagonal Enriched Image (Hex_E)

The hexagonal enriched image has a hexagonal pixel form on a hexagonal grid. The generation process is similar to the resampling process in [1], which has three steps: projecting the original image pixel intensities onto a grid of sub-pixels; estimating the values of subpixels at the resampling positions; estimating each new hexagonal pixel intensity in a new hexagonal arrangement. The three steps are elaborated in the following.

#### 3.1.1. A Grid of Virtual Image Sensor Pixels Is Constructed

**Figure 1:**
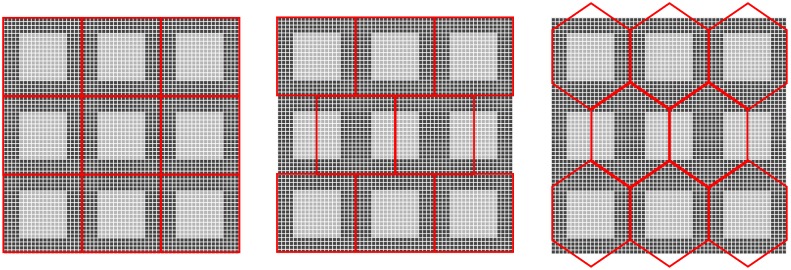
From left to right: the sensor rearrangement onto the subpixel, the projection of the square pixels onto the hexagonal grid by half pixel shifting method and the projection of the square pixels onto the hexagonal grid in generation of hexagonal image.

Each pixel is projected onto a grid of L × L square subpixels. By using the fill factor FF value, the size of the active area is defined as *S* × *S*, where $S=L\times \sqrt{FF}$. The intensity value of every pixel in the image sensor array is assigned to the virtual active area in the new grid. The intensities of subpixels in the non-sensitive areas are assigned to be zero. An example of such sensor rearrangement on a sub-pixel level is presented on the left in Figure 1, where there is a 3 × 3 pixel grid, and the light and dark grey areas represent the active and non-active areas in each pixel. Assuming L=30 and the active area is composed by 18 × 18 subpixels, and thereby the fill factor becomes 36% according to the above equation, and the intensities of active areas are represented by different grey level values. The size of the square subpixel grid for one pixel is examined from 20 × 20 to 40 × 40, the intensity in the generated images show no further significant changes after the size is 30 × 30. Thus, in the experiment, L is set to 30.

#### 3.1.2. The Second Step Is to Estimate the Values of Subpixels in the New Grid of Subpixels

Considering the statistical fluctuation of incident photons and their conversion to electrons on the sensor, a local Gaussian model is estimated by maximum likelihood method from each certain neighborhood area of pixels. Using each local model, a local noise source is generated and introduced to each certain neighborhood. Then inspired by Monte Carlo simulation, all subpixels in each certain neighborhood are estimated in an iteration process using the known pixel values (for sub-pixels in the active area) or by linear polynomial reconstruction (for subpixels in non-sensitive area). In each iteration step the number of subpixels of the active area in the actual pixel is varied from zero to total number of subpixels of active area (i.e., the total sub-pixel number is defined by the fill factor). By estimating the intensity values of the subpixels during the iteration process, a vector of intensity values for each subpixel is created from which the final subpixel value is optimally predicted using Bayesian inference method and maximum likelihood of Gaussian distribution.

#### 3.1.3. In the Third Step, the Subpixels Are Projected back to a Hexagonal Grid Shown as Red Grids on the Right of Figure 1, Where the Distance between Each Two Hexagonal Pixels Is the Same

Then the subpixels in each hexagonal area are estimated with respect to the virtual increase of the fill factor. The intensity value of a hexagonal pixel in the grid is the intensity value which has the strongest contribution in the histogram of belonging subpixels. The corresponding intensity is divided by the fill factor for removing the fill factor effect to obtain the hexagonal pixel intensity.

### 3.2. Generation of the Square Enriched Image (SQ_E)

The estimated square images are generated by three steps where the two steps explained in 3.1.1 and 3.1.2 are followed by a third step as follows. The subpixels are projected back to the original square grid shown as red grids on the left of Figure 1. The intensity value of each pixel in the square grid is the intensity value which has the strongest contribution in the histogram of its belonging subpixels. Then the corresponding intensity is divided by the fill factor to obtain the square pixel intensity by virtual increase of fill factor to 100% as the work in [1].

### 3.3. Generation of the Half Pixel Shift Image (HS) and Half Pixel Shift Enriched Image (HS_E)

The hexagonal grid in the previous work [3,4] is mimicked by a half-pixel shift which is derived from delaying sampling by half a pixel in the horizontal direction. The red grid, which is presented in the middle of Figure 1, is the new pseudohexagonal sampling structure whose pixel form is still square. The new pseudohexagonal grid is derived from a usual 2-D grid by shifting each even row a half pixel to the right and leaving odd rows unattached, or of course any similar translation. The half pixel shift image (HS) and half pixel shift enriched image (HS_E) are both generated from the original image (SQ) and enriched image (SQ_E) on the square grid, respectively.

## 4. Curviness Quantification

The curviness is quantified by comparison of the sharp transitions in contour of all correspondent objects in pair of images which have exact similar contents but two different contours; namely straight or curved contour. We define an image which has only straight or only curved contour as SC or CC image respectively. First and second order gradient operations are used in the quantification on each original image (i.e., SQ image type) and its set of generated images (i.e., Hex_E, SQ_E, HS_E, and HS image types). We elaborate these operations in following.

### 4.1. Implementing a First Order Gradient Operation

The familiar first order gradient ∇J is defined as:

(1) $$\nabla J\left(z\right)=J\left(z\right)-J\left(z^{\prime }\right)$$

where J represents the image, z and $z^{\prime }$ represent the positions of two adjacent pixels which have a common border; i.e., a common side or corner border. The angle between orientation of adjacent pixels and horizontal axis represents direction of the gradient. The first order gradient values of a pair of images (i.e., even with different grids) can be compared by computing and analyzing the eigenvalues and eigenvectors which are obtained by solving the expression $\left(A-\lambda_{j}I\right)e_{j}=0,$ where A is the 2 × *n* matrix with *n* number of first order gradient values of each of the images, λ and e are the eigenvalue and the eigenvector respectively, j is the index number with value of 1 or 2, and I is the identity matrix. In the comparison, when two images have same contents but different grids, the range of eigenvalues from small to large values indicate the similarity to dissimilarity between grid structures. When two images have the same content but different contours the curviness can be quantified by comparison of the eigenvalues related to the images.

### 4.2. Implementing Hessian Matrix on SQ, and SQ_E Images

Analyzing the second order gradient operation in form of Hessian matrix computation has an intuitive justification in the context of curvature quantification. The eigenvalues analysis of the Hessian extracts the principle gradient directions in which the local second order structure of an image is decomposed. This directly gives the direction of smallest curvature (along the contour) [26,27]. The Hessian matrix of:

(2) $$H=\left[\begin{array}{cc} J_{xx} & J_{xy}\\ J_{yx} & J_{yy} \end{array}\right]$$

is computed from convolution of the image J and gradients of the gaussian kernel $G=\frac{1}{2\pi \sigma^{2}}e^{-\frac{x^{2}+y^{2}}{2\sigma^{2}}}$ as follows:

$$J_{xx}=G_{xx}*J,\;J_{yy}=G_{yy}*J,\;J_{xy}=J_{yx}=G_{xy}*J,$$

where $G_{xx},\;G_{xy}$ and $G_{yy}$ represent the gradient kernel on the horizontal, vertical and diagonal directions, respectively.

The eigenvalues and eigenvectors of the Hessian matrix H are obtained by solving the expression $\left(H-\lambda_{j}^{hs}I\right)e_{j}^{hs}=0,$ where $\lambda_{j}^{hs}$ and $e_{j}^{hs}$ are the eigenvalue and the eigenvector, respectively, and j is the index number with value of 1 or 2. The first eigenvector (the one whose corresponding eigenvalue has the largest absolute value) is the direction of greatest curvature. The other eigenvector (always orthogonal to the first one) is the direction of least curvature. The corresponding eigenvalues are the respective amounts of these curvatures. Inspired by earlier work of Frangi et al. [22], three measurement parameters are derived from eigenvalues and eigenvectors of Hessian matrix which are used in comparison of the pair of images when they have the same content but different contours. The parameters are:

(3) $$P_{1}=arctang\left(\frac{e_{2x}^{hs}}{e_{2y}^{hs}}\right),$$

(4) $$P_{2}=\log\left(1+{\left(\frac{\lambda_{2}^{hs}\;}{\lambda_{1}^{hs}\;}\right)}^{2}\right)\;,$$

(5) $$P_{3}=\log\left(1+\lambda_{1}^{hs}{\;}^{2}+\lambda_{2}^{hs}{\;}^{2}\right),$$

where $\lambda_{1}^{hs}$ (the largest one) and $\lambda_{2}^{hs}$ are the eigenvalues of the Hessian matrix and $e_{1}^{hs}=\left[e_{1x}^{hs},e_{1y}^{hs}\right]$ $e_{2}^{hs}=\left[e_{2x}^{hs},e_{2y}^{hs}\right]$ are the related eigenvectors. $P_{1},\;P_{2},P_{3}$ measure the main orientation, the relation of the two principal curvatures (i.e., each of which measures amount of curvature bending in different directions), and second order structureness respectively. The dissimilarity of each pair of SC and CC images (i.e., which are having square grid and the same content but different contours) are measured by:

(6) $$D_{P_{j}}^{\left(SC,\;CC\right)}\;=\;\frac{var\;(P_{j}^{SC}\cdot \;P_{j}^{CC})}{var\;(P_{j}^{SC})*var\;(P_{j}^{CC})}$$

where SC and CC are a pair of images which have the same contents but different contours and the pair can be the type of SQ or SQ_E images, j is the index number (which can have values of 1, 2, or 3), $P_{j}$ is one of the three parameters according to the j, $P_{j}^{SC}$ and $P_{j}^{CC}$ are the measurement parameters $P_{j}$ applied on the pair of images of SC and CC, respectively.

### 4.3. Implementing Second Order Operation to Detect Saddle and Extremum Points

The second order gradient operation has been used to detect spatial critical points; i.e., saddle and extremum points. The number of critical points in an image depends on the contour shape. Thus, a pair of images with the same content but different contours can be compared using the detected critical points in each image. Generally, the critical points are detected when the gradient is zero. This means the Hessian matrix can be used in this relation; the critical points are found by using eigenvalues of the Hessian matrix on a square grid. However, on a square grid the zeros of gradient will in general not coincide with the grid points, but lie somewhere in between them. Kuiiper [28] showed that by converting the square grid to a hexagonal grid; implementing a half pixel shifting method, it is possible to detect a more accurate number of critical points in a square grid-based image. In his detection process based on hexagonal grida, each point has six neighbours. The sign of intensity difference for each of these neighbours with respect to the point itself is determined which results in its classification into four different points: regular, minimum or maximum (extremum), saddle, and degenerated saddle point. We used this detection process not only on square based grid images, but also on hexagonal grid based images to detect saddle and extremum points.

**Figure 2:**
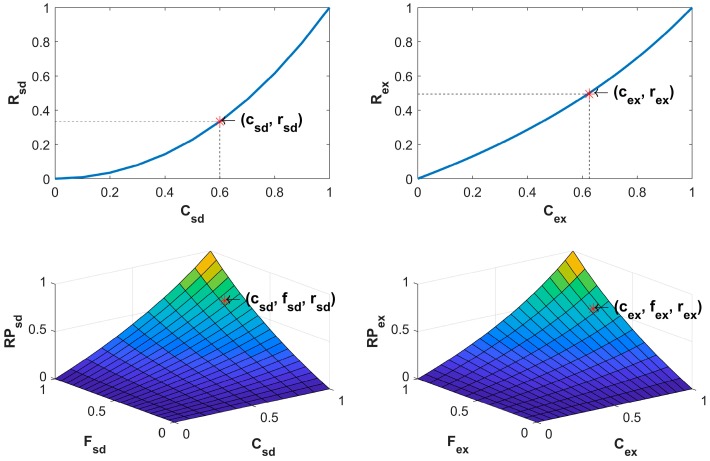
Typical characteristics of $R_{sd}\left(\;C_{sd}\;\right)$ in Equation (7), $R_{ex}\left(\;C_{ex}\;\right)$ in Equation (8), $RP_{sd}\left(C_{sd},F_{sd}\right)$ in Equation (9), $RP_{ex}\left(C_{ex},F_{ex}\right)$ in Equation (10) are shown in top left, top right, bottom left, and bottom right respectively. $C_{sd}$, $C_{ex}$, $F_{sd}$ , and $F_{ex}$ are sets of possible values of $c_{sd}$, $c_{ex}$, $f_{sd}$ , and $f_{ex}$, respectively.

In relation to curvature quantification, pair of SQ, SQ_E, and Hex_E image types are compared in relation to the detected critical points. The comparison measurement related to the saddle points is defined as:

(7) $$R_{saddle}=1-\;\frac{c_{sd}}{a_{sd}}\;\times \;\frac{c_{sd}}{\left|A_{sd}\cup B_{sd}\right|}\;\times \;\frac{b_{sd}}{a_{sd}},\;A_{sd}\leq \;B_{sd}$$

where $A_{sd}$ and $B_{sd}$ are two sets of $a_{sd}$ and $b_{sd}$ saddle points in images A and B respectively (where A and B are two types of images), $C_{sd}=\;A_{sd}\cap B_{sd}$ is the set of the saddle points that are on the same position in the two images with $c_{sd}=\;\left|C_{sd}\right|=\;\left|A_{sd}\cap B_{sd}\right|$ points, and $\left|A_{sd}\cup B_{sd}\right|$ is the number of all points which are in sets of $A_{sd}$ and $B_{sd}$. Equation (7) is a normalized nonlinear dissimilarity measurement function based on the common detected saddle points in the two images; see a typical of such a function characteristic in top left of Figure 2. The comparison measurement related to the extremum points is defined as:

(8) $$R_{extremum}=\;1-\;\frac{a_{ex}}{b_{ex}}\;\times \;\frac{c_{ex}}{\left|A_{ex}\cup B_{ex}\right|},\;\;\;B_{ex}\leq \;A_{ex}$$

where $A_{ex}$ and $B_{ex}$ are two sets of $a_{ex}$ and $b_{ex}$ extremum points where in images A and B, respectively (where A and B are two types of images), $C_{ex}=\;A_{ex}\cap B_{ex}$ is the set of the extremum points that are on the same position in the two images with $c_{ex}=\;\left|C_{ex}\right|=\;\left|A_{ex}\cap B_{ex}\right|$ points, and $\left|A_{ex}\cup B_{ex}\right|$ is all points which are in sets of $A_{ex}$ and $B_{ex}$. Equation (8) is a normalized nonlinear dissimilarity measurement function based on the common detected extremum points; see a typical of such a function characteristic in top right of Figure 2. The comparison measurement related to the saddle points between two pairs of images is defined as:

(9) $$RP_{saddle}=\;1-\frac{c_{sd}}{\left|A_{sd}\cup B_{sd}\right|}\;\times \;\frac{f_{sd}}{\left|D_{sd}\cup E_{sd}\right|}\;\times \;\frac{max\left(\begin{array}{cc} a_{sd}, & b_{sd} \end{array}\right)}{min\left(\begin{array}{cc} a_{sd}, & b_{sd} \end{array}\right)}\;\times \;\frac{max\left(\begin{array}{cc} d_{sd}, & e_{sd} \end{array}\right)}{min\left(\begin{array}{cc} d_{ex}, & e_{sd} \end{array}\right)}$$

where $a_{sd}$ $b_{sd}$ $d_{sd}$ and $e_{sd}$ are the numbers of saddle points in sets of $A_{sd}$ $B_{sd}$ $D_{sd}$ and $E_{sd}$ in image A, B, D and E, respectively. The two pairs of images are (A, D) and (B, E). Each pair of images has the same type and different type from another pair. The images A and B are the SC images where the images D and E are CC images. The $C_{sd}=\;A_{sd}\cap B_{sd}$ and $F_{sd}=D_{sd}\cap E_{sd}$ are the sets of the saddle points that are on the same position in each related two images with $c_{ex}=\;\left|C_{ex}\right|$ and $f_{ex}=\left|F_{ex}\right|$ points respectively. Equation (9) is a normalized 2D nonlinear dissimilarity measurement function based on the common detected saddle points in each pair of the images; see a typical of such a function characteristic in bottom left of Figure 2. The comparison measurement related to the extremum points between two pairs of images is defined as:

(10) $$RP_{extremum}=\;1-\;\frac{c_{ex}}{\left|A_{ex}\cup B_{ex}\right|}\;\times \;\frac{f_{ex}}{\left|D_{ex}\cup E_{ex}\right|}\;\times \;\frac{max\left(\begin{array}{cc} a_{ex}, & b_{ex} \end{array}\right)}{min\left(\begin{array}{cc} a_{ex}, & b_{ex} \end{array}\right)}\;\times \;\frac{max\left(\begin{array}{cc} d_{ex}, & e_{ex} \end{array}\right)}{min\left(\begin{array}{cc} d_{ex}, & e_{ex} \end{array}\right)}$$

where $a_{ex}$ $b_{ex}$ $d_{ex}$ and $e_{ex}$ are the numbers of extremum points in sets of $A_{ex}$ $B_{ex}$ $D_{ex}$ and $E_{ex}$ in image A, B, D and E, respectively. The two pairs of images are (A, D) and (B, E). Each pair of images has the same type and different type from another pair. The images A and B are the SC images where the images D and E are CC images. The $C_{ex}=\;A_{ex}\cap B_{ex}$ and $F_{ex}=D_{ex}\cap E_{ex}$ are the sets of the extremum points that are on the same position in each two related images with $c_{ex}=\;\left|C_{ex}\right|$ and $f_{ex}=\left|F_{ex}\right|$ points. Equation (10) is a normalized 2D nonlinear dissimilarity measurement function based on the common detected saddle points in each pair of the images; see a typical of such a function characteristic in bottom right of Figure 2.

## 5. Experimental Setup

**Figure 3:**
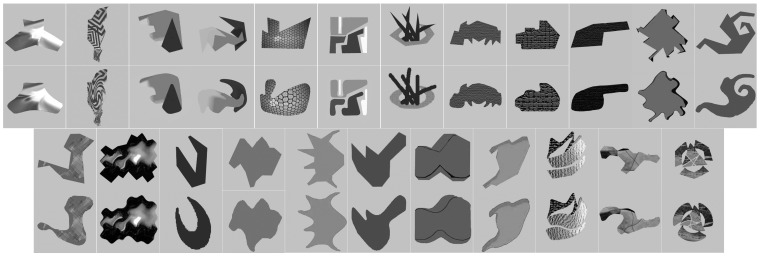
Twenty-three pairs of images from the database, where the images in first and third rows have sharp contours and the images in the second and fourth rows have the curved contours.

**Figure 4:**
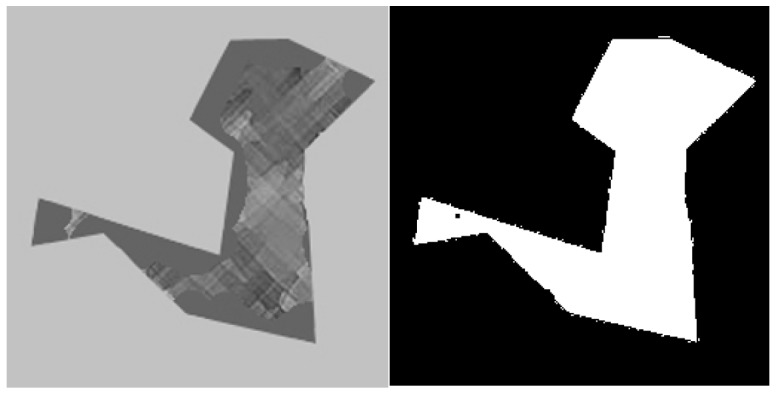
One of the database images and its template mask for detecting the background.

An image dataset from [29] is used for our experiments. The database was used earlier to investigate human visual preference of curved versus linear edges to find the physical elements in a visual stimulus which cause like or dislike of objects. The database is composed of 280 pairs of images, each pair of images have the same semantic meaning and general appearance. We found 51 image pairs had contour curvature differences additionally to the semantic meaning; i.e., straight to curved line. Then each pair of images with the same semantic content is defined as straight contour (SC) or curved contour (CC) images. Each of the images has the same resolution, 256 × 256, and in Uint8 format. Figure 3 shows twenty-three pairs of images which are randomly selected from the 51 pairs of images and used for the experiment. The images of the database are generated by computer graphic tools (i.e., without natural noise). However, they are compressed as jpg format in which noise is introduced accordingly as it is shown in Figure 3. It is certain that the noise affects the first and second order derivative operation of Equations (1) and (2). Some smoothing filters are used to reduce the noise but this significantly change the content of the object (straight or curved lines) in the images which is not appreciated due to the problematic goal (i.e., to compare straight lines vs curved ones). Instead of smoothing of the whole image, a template mask for each of the images is used to detect the background which is irrelevant to the object content, as it is shown in Figure 4. Each mask is generated automatically using morphological operations. When the images with different pixel structure and form are compared they are contaminated with the same amount of noise, thus effect of the noise on the results considered to be insignificant and the comparison results are obtained only from the masked area on the respective images. The images are converted to grayscale images, and then the fill factor of images is estimated by the method explained in [20]; fill factor value is estimated to be 36%. The impact of curved versus straight edges on the enriched hexagonal (Hex_E), enriched estimated square (SQ_E), enriched half pixel shifted (HS_E), and original (SQ) images is evaluated by computing the first and second order gradient operations; see Section 4.1 and Section 4.2, on the images. All images have the same resolution to ensure that the resolution is not affecting the number of gradients. All the processing is programmed and implemented by Matlab2017a on a stationary computer with an Intel i7-6850k CPU (Intel Corporation, California, USA, https://ark.intel.com/products/94188/) and a 32 GB RAM memory to keep the process stable and fast.

## 6. Results and Discussion

**Figure 5:**
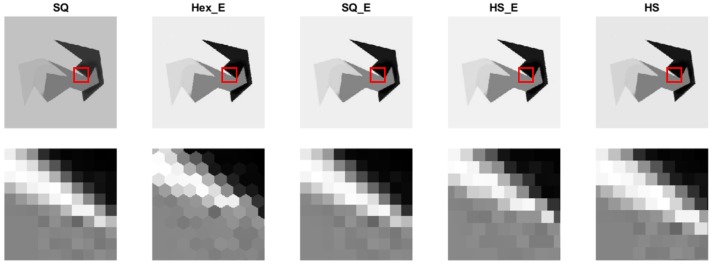
One of original images and its set of generated images.

One of the original images and the set of enriched related generated images; hexagonal, estimated square, and half-pixel shifted images, which were explained in Section 3 are shown in Figure 5. The images from left to right in the first row are the original image, and the related generated images. The images in the second row of Figure 5 are the zoomed region of the images (shown as red square). The generated images show better dynamic range in comparison to the original images, as it was shown in [21].

### 6.1. First Order Gradient Operation

**Figure 6:**
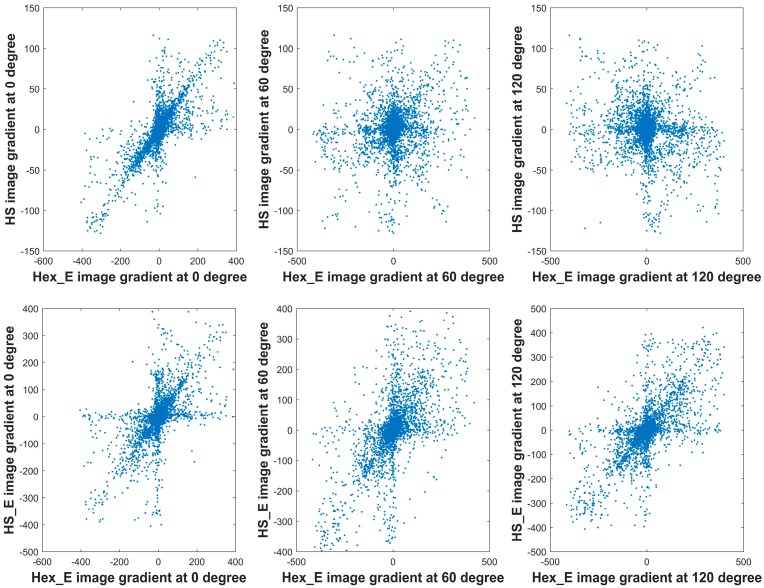

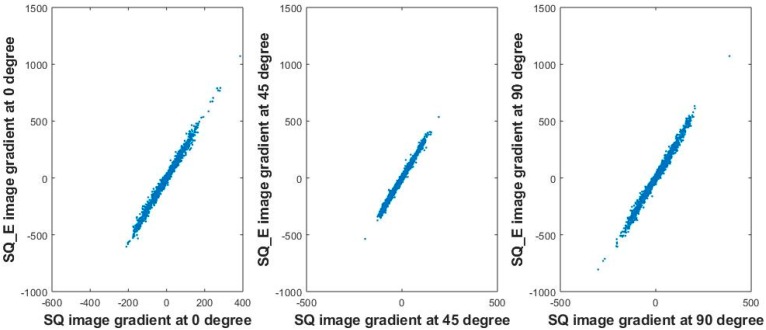
The gradients correlation between the Hex_E image (second column) and HS_E image (fourth column) shown in Figure 5 at directions of 0, 60 and 120 degrees (top row). And the gradients correlation between the SQ image (first column) and SQ_E image (third column) shown in Figure 5 at directions of 0, 45 and 90 degrees (bottom row).

The sharp transitions in the contour of each objects in the images—the curviness—is quantified by implementing the first and second order gradient operations on the pair of original images and their set of generated images; each operation process is explained in more details in Section 4. For the images on the hexagonal grid; hexagonal and half-pixel shift images, the first order gradients are computed at six directions, which are 0, 60, 120, 180, 240, and 300 degrees. Due to resolution similarity of the generated images the on hexagonal grid, their number of pixels and the computed gradient elements are the same. The top and middle row of Figure 6 shows the sorted first order gradient values from the generated Hex_E image (i.e., the image shown in Figure 5) in comparison to the generated HS and HS_E image at 0, 60 and 120 degrees from left to right respectively. The amount of spreading of the gradient values reveals the correlation between the grid structure of the images. The more similar the image grids are, the amount of spreading is less. The more densely the points are distributed, the less variation from the gradient results are expected. Due to the grid similarity of original images and the SQ_E images, the correlations of sorted gradient values at 0, 45 and 90 degrees between them are linear which are shown in the bottom row of Figure 6. However, the correlations of sorted gradient values at 0, 60 and 120 degrees on the pseudo hexagonal grid structure and hexagonal grid structure are nonlinear and dissimilar; shown in top and middle rows of Figure 6. 

**Figure 7:**
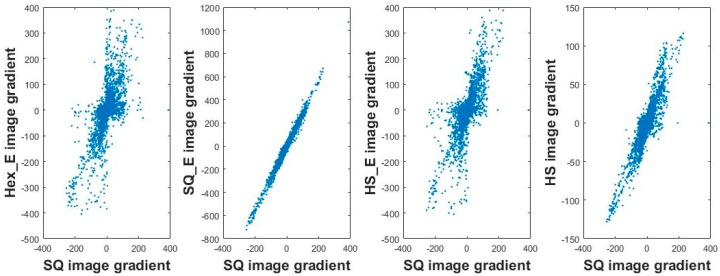
The gradient cross comparison at 0 degree between one of the original images and its generated images: Hex_E image (first), SQ_E image (second), HS_E image (third), and HS image (fourth).

The same is the correlation of sorted gradient values at 0 degree between the SQ image grid structure to both the pseudo hexagonal grid structure and hexagonal grid structure which are shown in Figure 7 on the first and third columns from left respectively; i.e., the correlation is in each case nonlinear and dissimilar.

Figure 7 shows that the gradient results from the four types of generated images in comparison to the original SQ image is different from each other; especially the second plot from left. This is because the grids in HS, HS_E and Hex_E images are more alike to each other and more different from the square grid (i.e., the grid of SQ and SQ_E images). The similarity/dissimilarity of each two grid structures are possible to visualize; as they are shown in Figure 6 and Figure 7. However, to quantify such a similarity/dissimilarity the first order gradient operation can be used as it is described in Section 4.1. Accordingly, the covariance of the gradient values of each two images are computed where each compared two images have the same contents but different grid structures. Then the eigenvalues and eigenvectors of each covariance matrix is computed using singular value decomposition (SVD) method.

**Figure 8:**
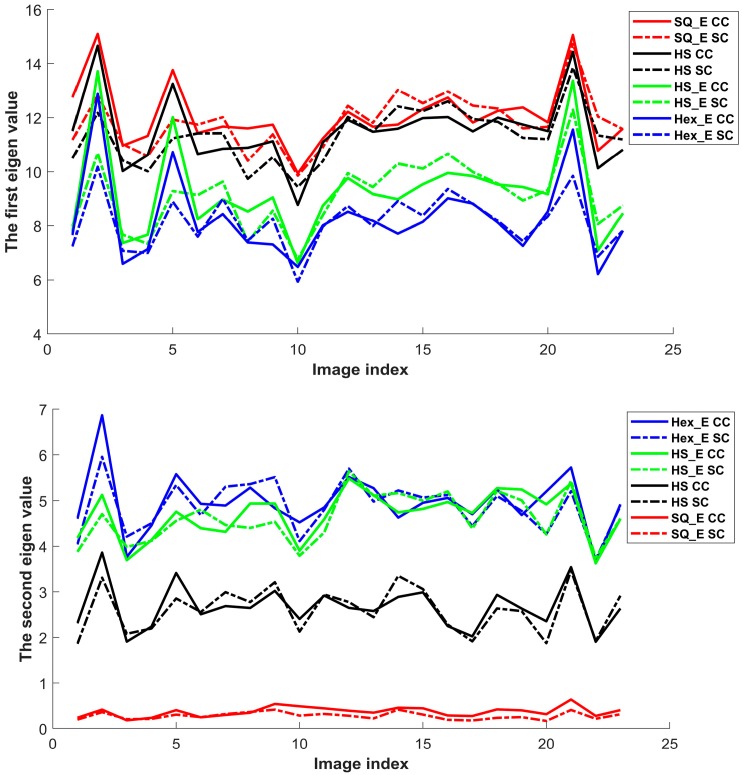
The first (**Top**) and second (**Bottom**) eigenvalues of the gradient values in a cross comparison between original image and four types of generated images. The blue lines represent the Hex_E images; the red lines represent the SQ_E images, the green lines represent the HS_E and the black lines represent the HS images. The continuous lines and dash lines represent the CC and SC images respectively.

The first eigenvalues, which are also the largest ones, of the covariance matrix between each pair of the original images and its set of generated images are shown in top of Figure 8, and the second eigenvalues are shown in the bottom of Figure 8. The blue, red, green and black lines represent the first or second eigenvalues of the covariance matrixes with respect to the four types of images presented in Section 3, respectively. The continuous lines and dash lines represent the computed first and second eigenvalues with respect to the original CC and SC images, respectively. Table 1 shows the summary of the comparison results of the two figures in Figure 8. The different properties among the types of images are caused by the diversity of their grid structure, pixel form or fill factor value. ‘Yes’ and ‘No’ in the table represent similarity and dissimilarity of such a property in relation between each generated image to the SQ image. The values in the last four columns of Table 1 are the sums of the first and second eigenvalues of the respected image type shown in Figure 8. In the table, the increase of the first or second eigenvalue indicates the increase of similarity or dissimilarity between the generated image and SQ image respectively. The SQ_E and HS images in relation to the SQ image show higher similarity than the other image types; see the first eigenvalue results in Table 1 and top figure in Figure 8. The comparison of these two types of images show that the grid structure is more important than pixel form and fill factor value to cause differences between them. The Hex_E and HS_E images in relation to the SQ image show higher dissimilarity, respectively. The comparison of these two types of images show that when the grid structures are the same the pixel form is more important than fill factor value to cause differences between them. The results show that the choice of grid structure, pixel form, and fill factor value are respectively important in generation of a new type of images. Here we should note that these three properties are not quite independent from each other. In Table 1, the results related to SC and CC for all four types of images show that they are clearly distinctive. However, the detail comparison of SC and CC in Figure 8 show that it is not possible to have a clear conclusion between SC and CC by first order operation; due to the results variation.

### 6.2. Hessian Matrix on SQ, and SQ_E Images

Table 2 and Table 3 show the measured $P_{1},\;P_{2},$ and $P_{3}$ parameters (Equations (3)–(5)) between SC and CC images having SQ or SQ_E image type using Hessian matrix; for more detail see Section 4.2. The bold result values in the tables show the higher one in comparison of each pair of SC and CC images. In Table 2, the results related to the CC images have higher values than SC results. $D_{P_{j}}^{\left(SC,CC\right)}\;\left(j=1,\;2,\;3\right)$ indicates that the contours in the CC images are distinctively different (have more curviness) than the ones in SC images. Table 3 shows the similar results, that the results values are higher in majority of CC images in respect to *SC* results; 94% of measurement values. Table 4 shows the dissimilarity measurement of $D_{P_{j}}^{\left(SC,CC\right)}\;\left(j=1,\;2,\;3\right)$ in Equation (7). The comparison between SQ and SQ_E images using $D_{P_{1}}^{\left(SC,CC\right)}$ and $D_{P_{2}}^{\left(SC,CC\right)}$ values is not conclusive due to undistinctive result values. However, using the $D_{P_{3}}^{\left(SC,CC\right)}$ values the comparison is possible due to clear distinctive results. According to this comparison the SQ_E images have in average 62.5% better performance than SQ images. As far as the dissimilarity of the $D_{P_{3}}^{\left(SC,CC\right)}$ measures the second order structureness, we can conclude that the SQ_E image type far better can perform in detection of curviness than SQ image type.

### 6.3. Saddle and Extremum Points

**Figure 9:**
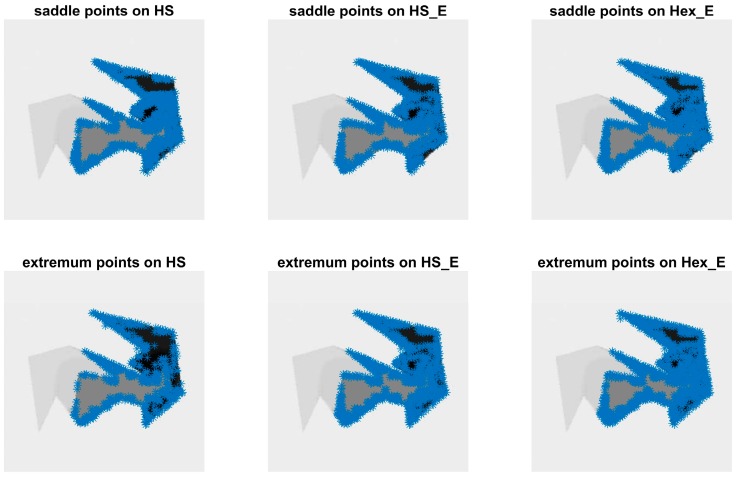
The detected saddle and extremum points on HS, HS_E and Hex_E images.

**Figure 10:**
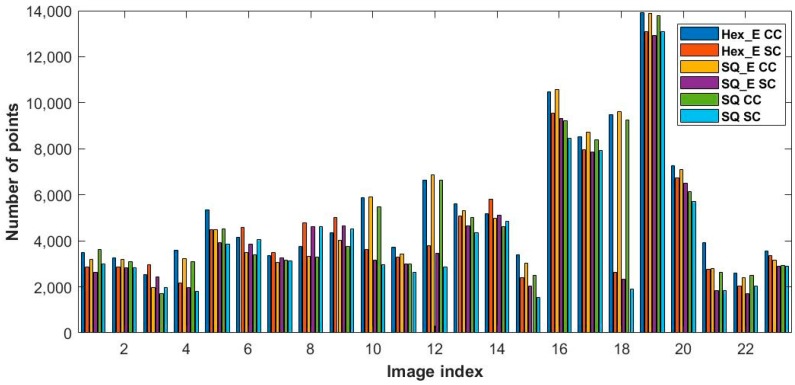
The number of the saddle and extremum points in each SC and CC image having Hex_E, SQ_E and SQ image types.

**Figure 11:**
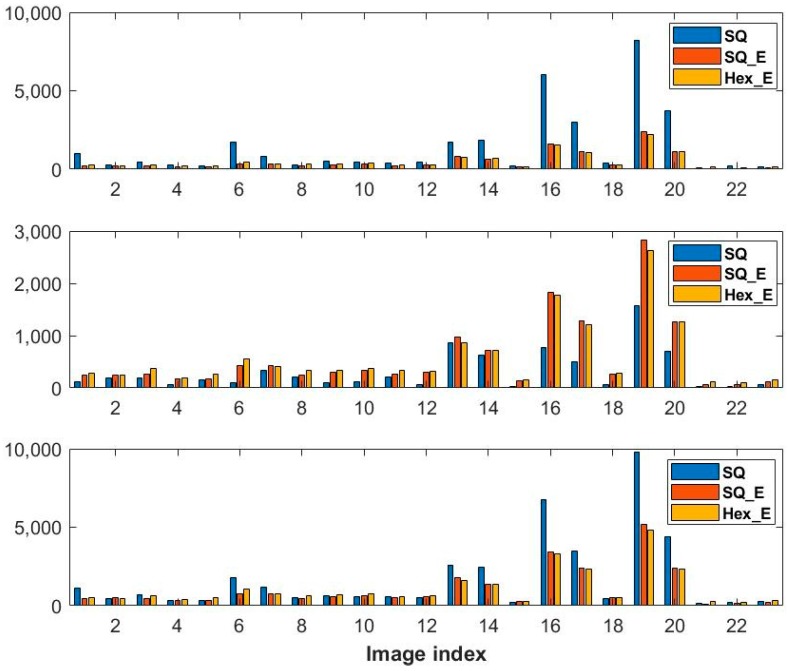
The number of the common saddle (**Top**), extremum points (**Middle**) and the critical points (**Bottom**) between SC and CC image pairs having Hex_E, SQ_E and SQ image types.

The detected saddle and extremum points from the second order gradient operation on the same HS, HS_E and Hex_E images shown in Figure 5 are shown in Figure 9, respectively. The HS and HS_E images are generated from its corresponding SQ and SQ_E images by converting the square grid to a hexagonal grid, implementing the half pixel shifting method proposed in Section 3.3. As it is discussed in Section 4.3, in comparison to SQ and SQ_E images the HS and HS_E images are better at detecting the critical points. The number of saddle and extremum points in each SC or CC image having Hex_E, SQ_E and SQ image types are shown in Figure 10. This shows that in 74% of pairs of CC and SC images of each image type, CC images have detected more critical points than SC images. However, in the figure the comparison results among Hex_E, SQ and SQ_E image types are still undistinctive. The top and middle figures in Figure 11 show that the number of common saddle and extremum points respectively, and the bottom figure shows the total number of the critical points. The common points are those points which are in the same position in each pair of SC and CC images. The results indicate that the SQ image type detects more common saddle points; in 87% of SC and CC image pairs, and Hex_E image type detects more extremum points; in 74% of image pairs. Due to that the critical points in SQ, SQ_E and Hex_E images are detected in the hexagonal grid; see Section 4.3, the difference of the results between image types is affected by having different pixel form and fill factor value. The results values between SQ_E and Hex_E image type in Figure 11 are close to each other; indicating that the pixel form has more effect on the second order gradient than the fill factor.

**Figure 12:**
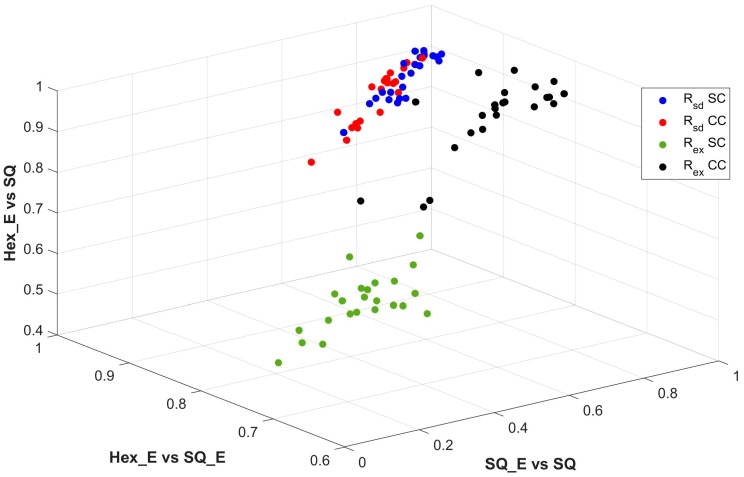
The co-relation of $R_{saddle}$ and $R_{extremum}$ values for SC and CC images from Table 5.

The normalized nonlinear dissimilarity measurement values of $R_{saddle}$ and $R_{extremum}$ for SC and CC from three pairs of comparisons between SQ_E, SQ and Hex_E images are computed by Equations (7) and (8) and shown in Table 5, where the higher value represents the larger dissimilarity of the contours between respected image types. For each pair of SC and CC, the larger value of $R_{saddle}$ or $R_{extremum}$ is shown bolded. The correlations of the three pairs of comparisons in Table 5 are shown in Figure 12, where the points of four colors of blue, red, green and black represent the correspondent values of $R_{saddle}$ and $R_{extremum}$ for each pair of SC and CC images respectively. In the figure, the three axes represent the three pairs of comparison between image types. The results of Table 5 show that the $R_{saddle}$ values for SC and CC images are close to each other, which is also verified in Figure 12; i.e., the blue points are mixed with red points. On the contrast, the $R_{extremum}$ values for SC and CC images in Table 5 are distinctive which is shown in Figure 12 by the green and black points. According to these results our conclusion is that only the extremum points can be used for quantifying the curviness; i.e., to distinguish a CC image from a SC image. Comparing the two comparisons of SQ_E & SQ and Hex_E & SQ_E and considering the three properties among the type of images; see Table 1, the measured dissimilarity in Table 5 for each of the comparisons is caused by fill factor and pixel form respectively. This is due to that SQ_E and SQ images are converted to hexagonal grid by half pixel shift for detection of critical points. Thus the grid structures of all three types of images in the two comparisons are the same. According to Table 5, the dissimilarity comparison values of the Hex_E & SQ_E are higher than the SQ_E & SQ, which shows that the pixel form is more important property than the fill factor to cause dissimilarity between images. This is consistent with the results which are presented in Table 1. Thus, from the results we concluded that the importance of the three properties from high to low is the grid structure, the pixel form and the fill factor, respectively.

Table 6 shows the results of the $RP_{saddle}$ and $RP_{extremum}$ values of three comparisons computed by the Equation (9) and Equation (10). The values in each column represent dissimilarity values based on the common detected saddle or extremum points in each pair of *SC* and *CC* images which is used to compare two different image types. The dissimilarity values in the table are not consistent for each two image types in comparison to previous results, indicating that processing SC and CC together is not an adequate way to quantify the difference between two image types. By combining the results in Table 5 and Table 6, we conclude that according to all the measurement the Hex_E image type has the largest dissimilarity in comparison to the other image types, which means the Hex_E is the best image type among our tested image types for quantifying the curviness of a contour.

## 7. Conclusions

In this paper, we present a software-based method to generate images with hexagonal pixel form on a hexagonal sensor grid. Each original rectangular pixel form is deformed to a hexagonal one using modelling of the incident photons onto the sensor surface. Four different image sensor forms and structures, including the proposed method, are evaluated by measuring their ability to detect curviness. We introduce a method for curviness quantification by comparison of the sharp transitions in contour of all correspondent objects in pair of images which have exact similar contents but two different contours. The quantification measurements are achieved by implementing first and second order gradient operations in form of several introduced and defined dissimilarity parameters. We show how first and second gradient operations, Hessian matrix computation, and measurement of the dissimilarity parameters can be implemented on both square and hexagonal grid structures. We pay special attention in detection of critical points (i.e., saddle and extremum points) using different image types.

The grid structure, pixel form and fill factor are proposed for representing the three major properties of the sensor characteristics and the results indicate that the grid structure is the most important one that makes difference between the type of images, and the pixel form is the second important one. The results of curviness quantification indicate that the detection of extremum points can be used to highly distinct CC from SC images. We show that enriched hexagonal image (i.e., Hex_E) is best in detection of curviness; according to its curviness measurement results, in comparison to the other tested image types. In the future, we intend to study other grid structures and pixel forms.

## Figures and Tables

**Table 1:** The summery of the comparison results of the two figures in Figure 8.

	Grid Structure	Pixel Form	Fill Factor	First Eigenvalue		Second Eigenvalue	
				SC	CC	SC	CC
SQ_E	Yes	Yes	No	272.98	277.86	6.43	8.51
HS	No	Yes	Yes	260.66	264.52	60.12	61.29
HS_E	No	Yes	No	210.19	212.98	106.33	107.89
Hex_E	No	No	No	187.28	190.26	112.32	114.14

**Table 2:** The measured $P_{1}$, $P_{2}$, $P_{3}$ parameters of SQ images using Hessian matrix.

Image Index	$P_{1}$		$P_{2}$		$P_{3}$	
	SC	CC	SC	CC	SC	CC
1	49.047	**50.953**	44.121	**55.879**	45.163	**54.837**
2	48.41	**51.59**	48.407	**51.593**	48.601	**51.399**
3	49.468	**50.532**	23.679	**76.321**	28.143	**71.857**
4	49.42	**50.58**	32.887	**67.113**	46.829	**53.171**
5	47.206	**52.794**	38.418	**61.582**	37.502	**62.498**
6	49.914	**50.086**	46.71	**53.29**	47.856	**52.144**
7	47.701	**52.299**	36.29	**63.71**	46.749	**53.251**
8	49.791	**50.209**	14.97	**85.03**	46.549	**53.451**
9	49.575	**50.425**	15.392	**84.608**	48.163	**51.837**
10	49.408	**50.592**	16.171	**83.829**	48.056	**51.944**
11	49.011	**50.989**	36.452	**63.548**	49.222	**50.778**
12	49.075	**50.925**	14.809	**85.191**	43.015	**56.985**
13	48.724	**51.276**	16.613	**83.387**	45.124	**54.876**
14	48.163	**51.837**	26.797	**73.203**	44.71	**55.29**
15	48.899	**51.101**	16.959	**83.041**	46.383	**53.617**
16	49.167	**50.833**	18.203	**81.797**	46.13	**53.87**
17	49.568	**50.432**	25.92	**74.08**	45.673	**54.327**
18	48.741	**51.259**	37.231	**62.769**	45.062	**54.938**
19	49.474	**50.526**	28.151	**71.849**	48.687	**51.313**
20	49.651	**50.349**	14.972	**85.028**	44.699	**55.301**
21	48.758	**51.242**	44.467	**55.533**	42.291	**57.709**
22	48.834	**51.166**	46.471	**53.529**	45.996	**54.004**
23	48.612	**51.388**	17.293	**82.707**	46.214	**53.786**

**Table 3:** The measured $P_{1}$, $P_{2}\;$, $P_{3}$ values of SQ_E images using Hessian matrix.

Image Index	$P_{1}$		$P_{2}$		$P_{3}$	
	SC	CC	SC	CC	SC	CC
1	49.353	**50.647**	44.349	**55.651**	44.976	**55.024**
2	49.337	**50.663**	48.76	**51.24**	48.343	**51.657**
3	**51.625**	48.375	**66.592**	33.408	30.413	**69.587**
4	**50.057**	49.943	30.517	**69.483**	43.874	**56.126**
5	47.565	**52.435**	37.025	**62.975**	35.033	**64.967**
6	**50.474**	49.526	48.374	**51.626**	47.124	**52.876**
7	48.212	**51.788**	36.412	**63.588**	44.62	**55.38**
8	49.627	**50.373**	15.485	**84.515**	41.143	**58.857**
9	49.341	**50.659**	15.857	**84.143**	47.783	**52.217**
10	49.185	**50.815**	16.628	**83.372**	47.98	**52.02**
11	49.821	**50.179**	34.757	**65.243**	41.404	**58.596**
12	48.961	**51.039**	15.126	**84.874**	43.718	**56.282**
13	48.858	**51.142**	19.546	**80.454**	45.803	**54.197**
14	48.583	**51.417**	32.959	**67.041**	45.682	**54.318**
15	49.016	**50.984**	17.626	**82.374**	46.96	**53.04**
16	49.262	**50.738**	23.977	**76.023**	46.319	**53.681**
17	49.564	**50.436**	25.45	**74.55**	44.955	**55.045**
18	48.858	**51.142**	35.824	**64.176**	44.511	**55.489**
19	49.473	**50.527**	33.351	**66.649**	47.252	**52.748**
20	49.399	**50.601**	19.883	**80.117**	44.592	**55.408**
21	48.437	**51.563**	43.321	**56.679**	41.176	**58.824**
22	49.108	**50.892**	46.233	**53.767**	44.769	**55.231**
23	48.605	**51.395**	17.534	**82.466**	46.788	**53.212**

**Table 4:** The dissimilarity measurement of $D_{P_{j}}^{\left(SC,CC\right)}\;\left(j=1,\;2,\;3\right)$ for SQ and SQ_E image types using Hessian matrix.

Image Index	SQ			SQ_E		
	$D_{P_{1}}^{SC,CC}$	$D_{P_{2}}^{SC,CC}$	$D_{P_{3}}^{SC,CC}$	$D_{P_{1}}^{SC,CC}$	$D_{P_{2}}^{SC,CC}$	$D_{P_{3}}^{SC,CC}$
1	9.475	12.085	19.567	8.504	11.644	31.7
2	5.942	10.548	10.423	5.117	10.378	19.319
3	11.581	13.929	18.852	10.244	13.112	28.209
4	13.036	11.043	16.944	12.121	11.14	27.646
5	6.784	8.598	7.894	6.535	8.884	14.992
6	8.807	11.69	14.253	7.345	11.121	23.045
7	9.523	9.535	12.147	8.68	9.189	20.22
8	5.967	9.278	9.236	5.789	9.207	17.652
9	6.01	10.419	8.565	6.001	10.462	15.91
10	5.019	9.401	9.651	4.979	9.293	18.443
11	12.695	9.951	11.685	11.886	9.932	19.111
12	7.973	10.289	11.85	7.81	10.494	18.112
13	6.344	13.968	13.87	6.164	12.58	23.444
14	3.615	11.937	12.011	3.496	9.546	19.285
15	10.371	11.426	11.641	9.43	11.762	15.419
16	9.994	15.302	17.45	9.42	14.25	25.318
17	8.455	14.049	17.837	8.149	14.028	28.25
18	6.252	12.369	14.174	6.28	12.709	19.231
19	7.297	11.696	11.081	6.889	9.639	15.6
20	8.232	15.834	17.783	7.755	14.707	29.075
21	6.396	8.167	7.962	6.236	8.013	15.206
22	11.36	10.291	16.176	10.966	10.115	28.117
23	5.759	10.059	9.716	5.709	10.14	17.785

**Table 5:** The $R_{saddle}$ and $R_{extremum}$ values for SC and CC between SQ_E, SQ and Hex_E image types.

Image Index	SQ_E & SQ				Hex_E & SQ_E				Hex_E & SQ			
	$R_{saddle}$		$R_{extremum}$		$R_{saddle}$		$R_{extremum}$		$R_{saddle}$		$R_{extremum}$	
	SC	CC	SC	CC	SC	CC	SC	CC	SC	CC	SC	CC
1	**0.865**	0.754	0.299	**0.812**	0.940	**0.934**	0.769	**0.814**	**0.946**	0.916	0.596	**0.922**
2	**0.700**	0.690	0.394	**0.499**	0.912	**0.922**	0.710	**0.75**	0.909	**0.918**	0.688	**0.781**
3	**0.866**	0.836	0.343	**0.868**	**0.941**	0.928	0.756	**0.866**	**0.957**	0.954	0.610	**0.954**
4	**0.857**	0.820	0.329	**0.828**	**0.948**	0.940	0.739	**0.811**	**0.953**	0.938	0.620	**0.926**
5	**0.690**	0.538	0.328	**0.385**	**0.896**	0.890	0.701	**0.778**	**0.923**	0.910	0.668	**0.801**
6	**0.786**	0.756	0.277	**0.915**	**0.929**	0.919	0.767	**0.841**	0.892	**0.893**	0.538	**0.967**
7	**0.836**	0.731	0.340	**0.792**	**0.931**	0.930	0.735	**0.802**	**0.933**	0.899	0.638	**0.909**
8	0.528	**0.542**	0.342	**0.510**	**0.876**	0.867	0.680	**0.747**	0.871	**0.896**	0.649	**0.797**
9	**0.886**	0.769	0.515	**0.792**	**0.934**	0.933	0.763	**0.821**	**0.943**	0.902	0.698	**0.896**
10	**0.631**	0.524	0.340	**0.865**	0.894	**0.919**	0.662	**0.786**	**0.908**	0.770	0.613	**0.926**
11	0.807	**0.824**	0.292	**0.729**	0.922	**0.932**	0.707	**0.804**	**0.948**	0.939	0.623	**0.878**
12	**0.877**	0.687	0.273	**0.898**	0.926	**0.940**	0.734	**0.786**	**0.94**	0.805	0.577	**0.942**
13	**0.782**	0.757	0.260	**0.749**	0.928	**0.939**	0.739	**0.798**	**0.921**	0.907	0.608	**0.886**
14	**0.855**	0.828	0.324	**0.871**	0.940	**0.949**	0.741	**0.831**	**0.943**	0.917	0.602	**0.928**
15	**0.893**	0.877	0.321	**0.830**	0.930	**0.947**	0.725	**0.809**	**0.950**	0.944	0.583	**0.929**
16	**0.790**	0.744	0.114	**0.940**	0.926	**0.938**	0.751	**0.798**	**0.866**	0.833	0.478	**0.964**
17	**0.757**	0.698	0.149	**0.900**	0.933	**0.942**	0.737	**0.783**	**0.865**	0.818	0.530	**0.944**
18	**0.877**	0.654	0.280	**0.921**	0.934	**0.938**	0.729	**0.774**	**0.947**	0.784	0.583	**0.954**
19	**0.765**	0.671	0.193	**0.890**	0.925	**0.940**	0.731	**0.772**	**0.861**	0.810	0.522	**0.937**
20	**0.833**	0.803	0.187	**0.906**	0.941	**0.948**	0.761	**0.807**	**0.908**	0.889	0.537	**0.952**
21	0.683	**0.709**	0.308	**0.704**	0.879	**0.910**	0.692	**0.868**	0.920	**0.932**	0.620	**0.915**
22	**0.813**	0.784	0.349	**0.828**	0.941	**0.951**	0.775	**0.822**	**0.937**	0.899	0.673	**0.905**
23	0.656	**0.759**	0.319	**0.671**	0.898	**0.932**	0.685	**0.796**	0.913	**0.931**	0.620	**0.859**

**Table 6:** The $RP_{saddle}$ and $RP_{extremum}\;$ values of three comparisons computed by the Equation (9) and Equation (10).

Image Index	SQ_E vs. SQ		Hex_E vs. SQ_E		Hex_E vs. SQ	
	$RP_{saddle}$	$RP_{extremum}$	$RP_{saddle}$	$RP_{extremum}$	$RP_{saddle}$	$RP_{extremum}$
1	0.885	0.505	0.963	0.945	0.959	0.850
2	0.776	0.630	0.949	0.919	0.947	0.907
3	0.907	0.567	0.961	0.942	0.974	0.843
4	0.899	0.518	0.967	0.934	0.969	0.841
5	0.714	0.525	0.933	0.901	0.950	0.886
6	0.850	0.516	0.954	0.945	0.932	0.819
7	0.868	0.555	0.958	0.932	0.951	0.863
8	0.638	0.567	0.919	0.894	0.926	0.875
9	0.893	0.727	0.960	0.948	0.954	0.897
10	0.708	0.555	0.942	0.927	0.913	0.856
11	0.878	0.538	0.955	0.920	0.967	0.845
12	0.880	0.459	0.960	0.942	0.939	0.825
13	0.845	0.492	0.960	0.939	0.948	0.858
14	0.900	0.608	0.967	0.953	0.959	0.862
15	0.929	0.512	0.963	0.927	0.969	0.826
16	0.847	0.260	0.960	0.950	0.906	0.738
17	0.821	0.323	0.963	0.943	0.902	0.791
18	0.875	0.407	0.961	0.938	0.940	0.802
19	0.818	0.379	0.960	0.936	0.898	0.781
20	0.882	0.359	0.967	0.950	0.938	0.794
21	0.785	0.560	0.935	0.920	0.955	0.864
22	0.868	0.623	0.967	0.954	0.951	0.880
23	0.794	0.569	0.948	0.913	0.953	0.860
